# Carbon nanoparticles suspension injection for photothermal therapy of xenografted human thyroid carcinoma *in vivo*

**DOI:** 10.1002/mco2.28

**Published:** 2020-09-10

**Authors:** Yuanfang Huang, Guangfu Zeng, Qian Xin, Jinmei Yang, Cheng Zeng, Kexin Tang, Sheng‐Tao Yang, Xiaohai Tang

**Affiliations:** ^1^ Sichuan Enray Pharmaceutical Sciences Company Chengdu P. R. China; ^2^ College of Chemistry and Environment Protection Engineering Southwest Minzu University Chengdu P. R. China

**Keywords:** carbon nanoparticles, photothermal therapy, temperature control, theranostics, tumor

## Abstract

Due to the unique structure, carbon nanomaterials could convert near‐infrared (NIR) light into heat efficiently in tumor ablation using photothermal therapy (PTT). Carbon nanoparticles suspension injection (CNSI) is a commercial imaging reagent for lymph node mapping. CNSI has similar structural characteristics to other carbon nanomaterials, and thus, might be applied as photothermal agent. Herein, we evaluated the photothermal conversion ability and therapeutic effects of CNSI on thyroid carcinoma. CNSI was composed by carbon nanoparticle cores and polyvinylpyrrolidone K30 as the dispersion reagent. CNSI absorbed NIR light efficiently following the Lambert‐Beer law. The temperature of CNSI dispersion increased quickly under the NIR irradiation. CNSI killed the TCP‐1 thyroid carcinoma cells under 808 nm laser irradiation at 0.5 W/cm^2^, while CNSI or NIR irradiation treatment alone did not demonstrate this effect. Temperature increases were observed in tumor injected with CNSI under NIR irradiation. After three irradiation treatments, the tumor growth was completely blocked and the disruption of cellular structure was observed. When the tumor temperatures reached 53^°^C during treatment, the tumors did not recur within the observation period of 3 months. Our results suggested that CNSI might be used for PTT through “off label” use to benefit the patients immediately.

## Graphical abstract

Carbon nanoparticles suspension injection (CNSI) has excellent photothermal conversion ability for photothermal therapy of thyroid carcinoma. When the tumor temperatures reached 53^°^C during treatment, the tumors did not recur within the observation period of 3 months.

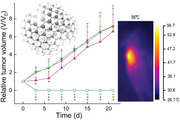

## 1 INTRODUCTION

Photothermal therapy (PTT) generates sufficient heat under near‐infrared (NIR) light irradiation with the help of photothermal conversion agents (PTAs), which has found great potential in tumor ablation.^1^, ^2^ PTT has many advantages, such as noninvasive treatment, minimal side effects, and high specificity. In terms of PTT research, the most important current issue is designing better PTA. Ideally, a PTA should have large absorption cross‐section, excellent photothermal conversion efficiency, good photothermal stability, low toxicity, and proper functionalities.^1^, ^2^ To this regard, organic molecules,^3^ polymers,^4^ nanomaterials,^5^ and metal‐organic frameworks^6^ are widely studied and adopted as PTAs.

Among the most promising PTAs, carbon nanomaterials have attracted tremendous interest.^7^, ^8^ The sp^2^ domains in carbon nanomaterials absorb NIR light efficiently to excite and induce surface plasmons.^9^ Then, the transmitting random dipoles and resonance were finally converted into thermal photon energy output. Therefore, carbon nanomaterials have high photothermal performance in PTT. For examples, Lu et al modified carbon nanotubes (CNTs) with polyethylene glycol, Cy7, and insulin‐like growth factor receptor for optical imaging guided PTT of orthotopic pancreatic cancer.^10^ Li et al prepared erythrocyte membrane camouflaged graphene oxide for photothermal chemotherapy, where the heat was able to promote the release of doxorubicin (DOX) and ablate the tumor.^11^ Dong et al found that bamboo charcoal nanoparticles had good photothermal performance and could be used as NIR responsive drug carrier of DOX for the chemotherapy/photothermal dynergistic therapy.^12^ Despite the encouraging achievements, there are two major issues hindering the clinical uses of these carbon nanomaterials. First, the aforementioned carbon nanomaterials are too complicated to be uniform. The batch‐to‐batch variations are too big for clinical applications.^13^ Second, there is great concern regarding their biosafety. The adsorption, distribution, metabolism, excretion and toxicity (ADME/T) of carbon nanomaterials are distinct from traditional small‐molecule drugs.^14^, ^15^ Therefore, these newly developed cancer nanomedicines have not been applied clinically.

Carbon nanoparticle suspension injection (CNSI, trade name: Canarine) is the only commercialized and clinically applied carbon nanomaterials, which is used to stain the tumor drainage lymph node black after intratumoral injection.^16^, ^17^ CNSI has been applied in surgical procedures on advanced gastric cancer,^18^ breast cancer,^19^ and papillary thyroid carcinoma.^20^ Presently, over 100 000 patients per year received CNSI injection during oncological surgery. Experimental evaluations and clinical observations have collectively confirmed the biosafety of CNSI.^21^, ^22^, ^23^ Therefore, unlike other carbon nanomaterials, when developing new clinical applications, CNSI could be immediately accessible for cancer patients through “off label” use.

CNSI has the similar structural characteristics to other carbon nanomaterials and may possess similar photothermal conversion capability for PTT applications.^7^, ^8^, ^9^ Herein, we evaluated the photothermal conversion and therapeutic efficacy of CNSI for tumor therapy both *in vitro* and *in vivo*. CNSI was characterized using multiple techniques. The absorbance of CNSI in NIR I window was recorded. The temperature increases of CNSI under 808 nm irradiation were measured. The inhibition of TPC‐1 thyroid cancer cells by CNSI under NIR irradiation was evaluated. The tumor ablation by CNSI under NIR irradiation was performed in tumor bearing mice. The structural disruption of tumor tissues was investigated by hematoxylin‐eosin (HE) staining. The implication of CNSI for cancer PTT is discussed.

## 2 RESULTS AND DISCUSSION

### 2.1 Characterization of CNSI

**FIGURE 1:**
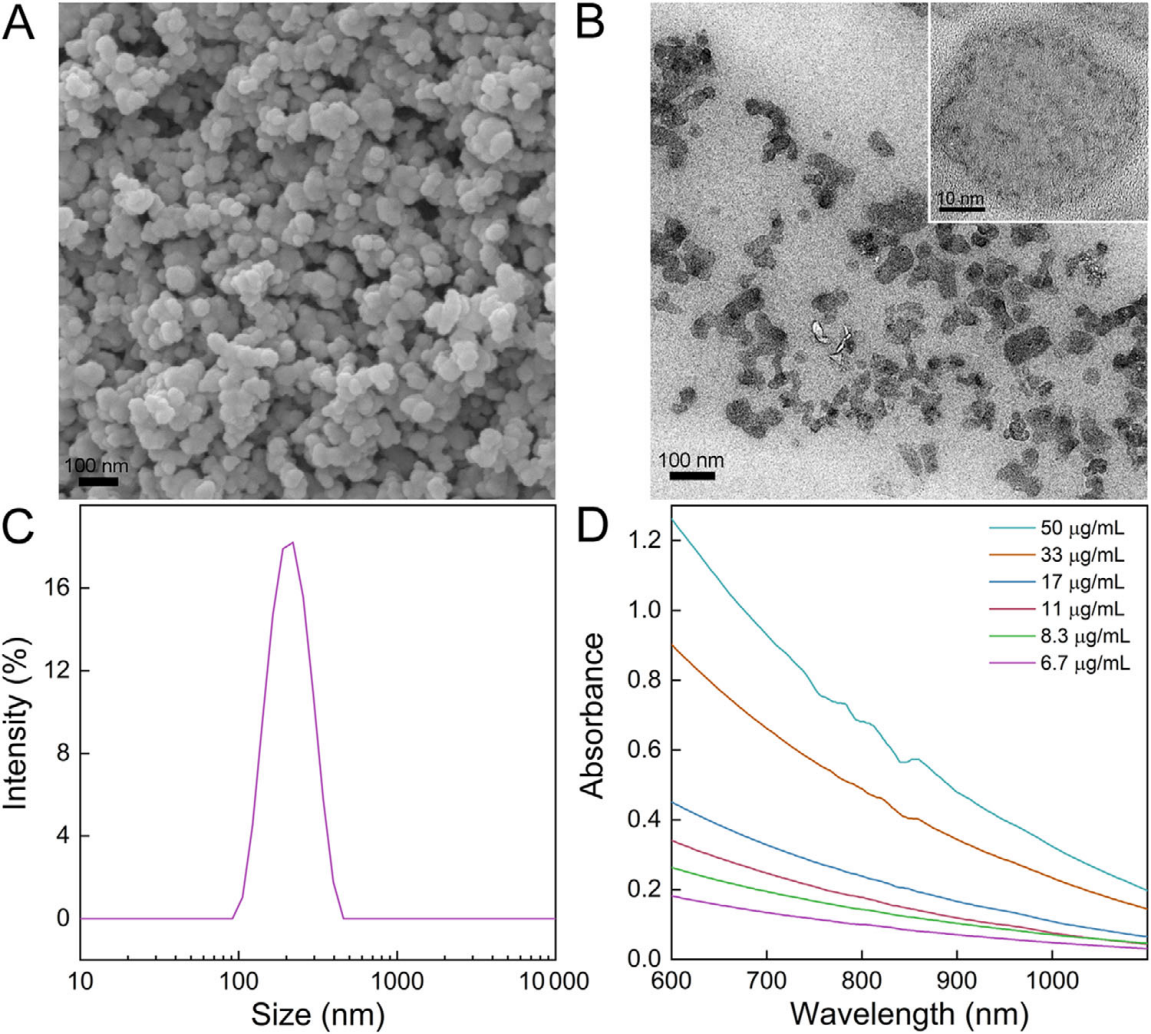
Characterization of CNSI. (A) SEM image of the starting material CH40 ash; (B) TEM image of CNSI with an inset of higher magnification; (C) DLS spectrum of CNSI; and (D) vis‐NIR absorbance spectrum of CNSI

The starting material used for CNSI was CH40 carbon ash. Under scanning electron microscope (SEM), the small particles of approximately 20‐30 nm in diameter were clearly recognized (Figure 1A). The main element of these carbon particles is carbon. According to X‐ray photoelectron spectroscopy (XPS) analysis, there were 97.0% of C, 1.9% of O, 0.9% of N, and 0.2% of S in the carbon particles. The C1s XPS spectrum indicated that there were sp^2^ and sp^3^ carbon atoms in carbon particles (Figure S1A), where the sp^2^ domains were also reflected by the G band in Raman spectroscopy (Figure S1B). The presence of sp^2^ carbon in carbon nanomaterials was widely reported in the literature.^24^ Upon the addition of polyvinylpyrrolidone K30 (PVP), the carbon particles became well dispersed, forming CNSI after homogenization. Dispersible particles were observed under transmission electron microscope (TEM). Even at high magnification, it was impossible to recognize the crystallinity (Figure 1B), very similar to carbon quantum dots.^25^ According to X‐ray diffraction (XRD) spectrum, there was graphite carbon in CNSI (Figure S2A). In the IR spectra, the presence of ‐OH group was evidenced at 3446 cm^−1^ peak (Figure S2B). C‐H bond was found at 2926 cm^−1^. The graphite carbon (C = C) was indicated at 1643 cm^−1^. The C‐N of PVP was found at 1282 cm^−1^, which was not found in CH40 ash (carbon nanoparticles without PVP coating). These particles were slightly aggregated in solution according to the dynamic light scattering (DLS) measurement, where the main peak occurred at 190 nm (Figure 1C). The good dispersion was due to the polymer chains rather than the electrostatic repulsion, because the zeta potential was only 14.0 mV. CNSI showed concentration‐dependent light absorption at NIR I window (Figure 1D), similar to other carbon nanomaterials.^24^ The absorbance at 808 nm followed the Lambert‐Beer law (Figure S3). These data indicated that CNSI could absorb NIR light, which might be applied in PTT.

### 2.2 *In vitro* photothermal effect

**FIGURE 2:**
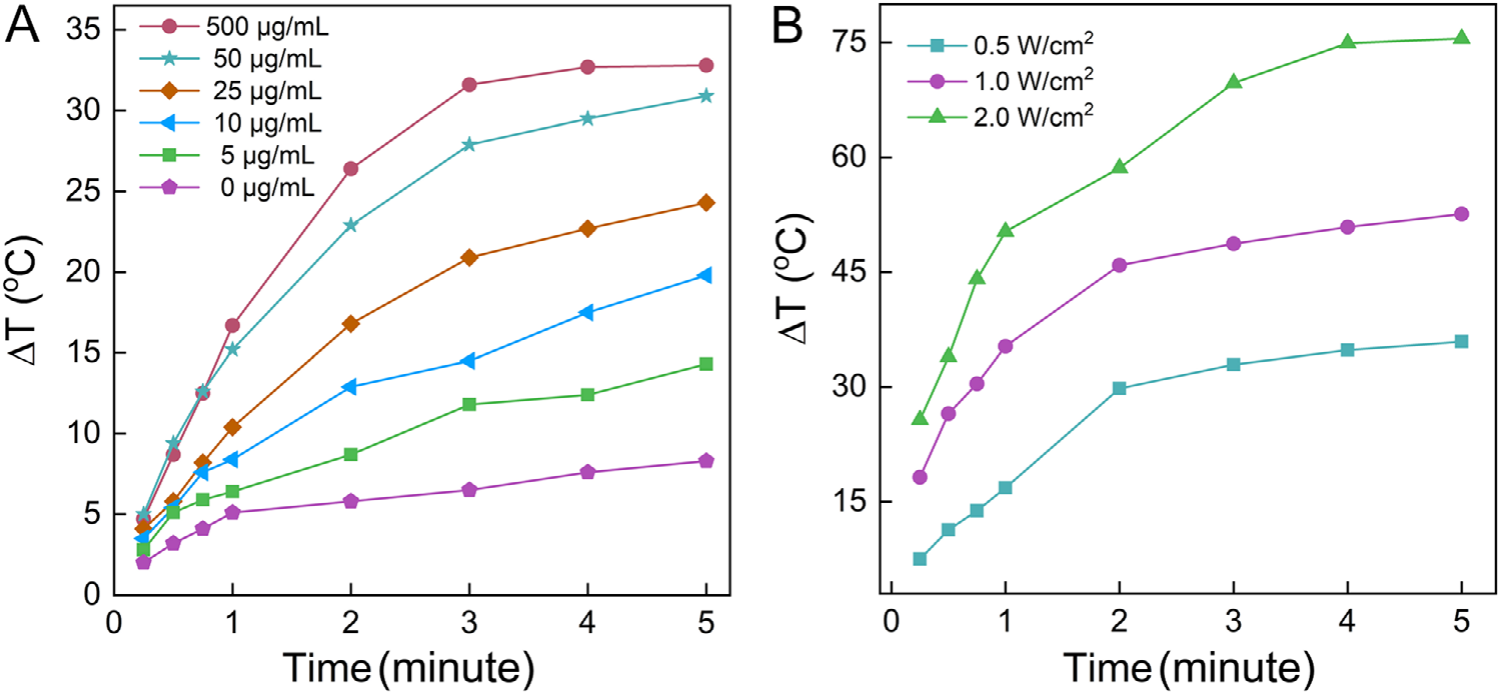
Representative plots for *in vitro* heating experiments with CNSI dispersion: (A) concentration‐dependent heating and (B) irradiation power density‐dependent heating

To verify the photothermal conversion capability of CNSI, we measured the temperature increases of CNSI dispersion under NIR irradiation *in vitro*. During the 5‐minute irradiation at 0.5 W/cm^2^, the blank solution showed that very slow temperature increases within 1 minute and the final increase was 8.3^°^C (Figure 2A). With the inclusion of carbon nanoparticles, 5 µg/mL of the CNSI dispersion showed a faster temperature increase, with a final temperature increase of 14.3^°^C. The higher CNSI concentration resulted in greater temperature increases. At 50 µg/mL, the final temperature increase of the dispersion reached 30.9^°^C. Further increases in CNSI concentration beyond this point did not significantly enhance the photothermal effect, which might be due to the NIR light already being fully absorbed. The final temperature increase of the dispersion was 32.8^°^C at 500 µg/mL. In another set of evaluation, the higher irradiation power density led to the faster temperature increase (Figure 2B). At 2 W/cm^2^, the final temperature increase of the dispersion reached 75.5^°^C, suggesting that CNSI dispersions possess efficient photothermal conversion capability, competitive to other carbon nanomaterials. For instance, graphene quantum dots of 0.3 mg/mL showed a temperature increase of about 40^°^C after 5‐minute irradiation at 1.0 W/cm^2^@1064 nm.^26^ The dispersion of 0.5 mg/mL Fe_3_O_4_ modified graphene was heated from 20^°^C to approximately 60^°^C after 10‐minute irradiation at 1.0 W/cm^2^@805 nm.^27^ Beyond the efficient photothermal conversion capability, CNSI was stable against the laser irradiation according to DLS measurements. The particle size decreased less than after 5‐minute irradiation (Figure S4). The excellent performance of CNSI in photothermal conversion might allow the applications in PTT.

### 2.3 *In vitro* therapeutic effect of CNSI under NIR irradiation

**FIGURE 3:**
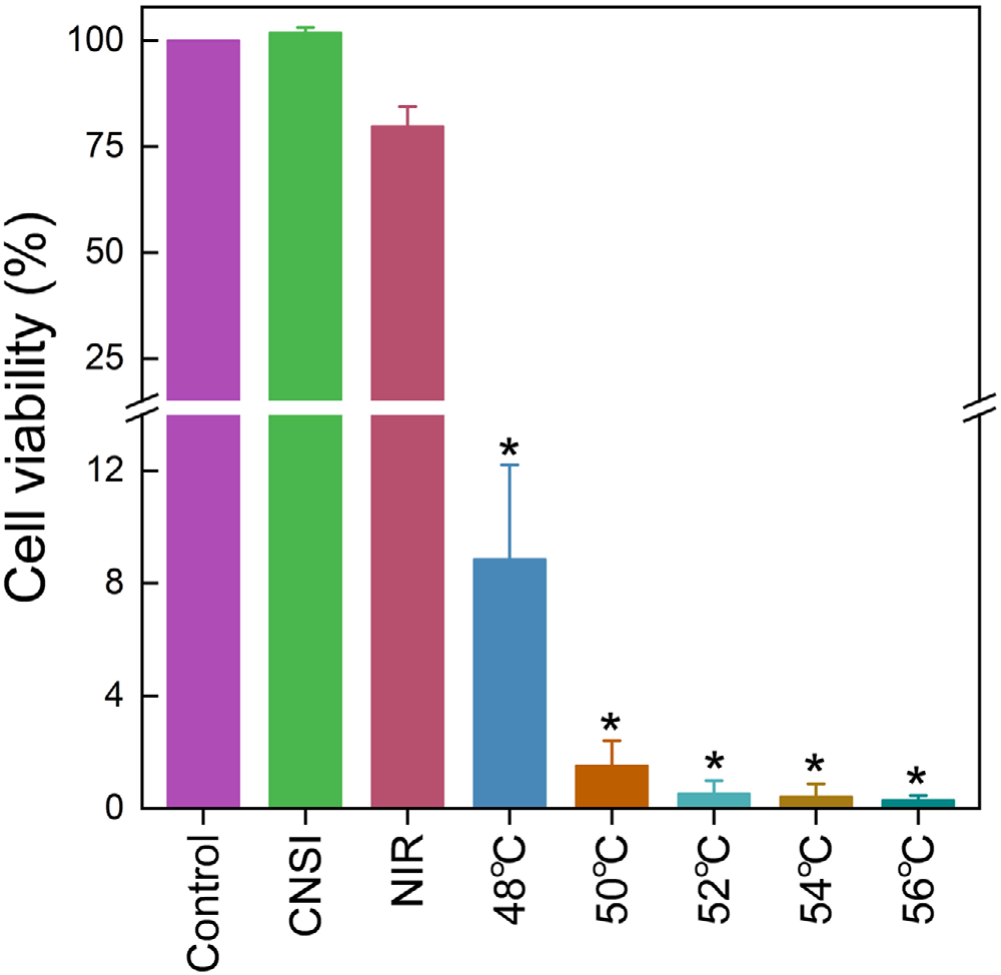
Cell viability of TPC‐1 cells after CNSI+NIR treatment at different temperatures. * *P* < .05 compared with control group (*n *= 3)

To verify the therapeutic effect of CNSI in PTT, we measured the cell viability changes of thyroid cancer TPC‐1 cells (Figure 3). According to the concentration‐dependence evaluation, 5 and 10 µg/mL CNSI were not efficient for PTT (Figure S5A). At 50 and 100 µg/mL, most cells were killed after CNSI+NIR exposure. Separately, CNSI alone was nontoxic to TPC‐1 cells without NIR irradiation (Figure S5B). Because CNSI at 50 µg/mL was sufficient enough in photothermal conversion, so CNSI at 50 µg/mL was adopted in the *in vitro* therapy. NIR irradiation led to slight toxicity due to the small photothermal effect and the viability decreased to 79.8% of the control. In the presence of CNSI as PTA, the temperature increases were much larger, so the cell viability loss became obvious. When the heating temperature was maintained at 48^°^C, the viability sharply decreased to 8.9% of the control, corresponding to the loss of 91.1%. The cell viability levels were less than 1% of control at temperatures of 52^°^C and higher. Together, these results suggested that CNSI has potential for use as a PTA during cancer cells.

### 2.4 *In vivo* tumor ablation by CNSI under NIR irradiation

**FIGURE 4:**
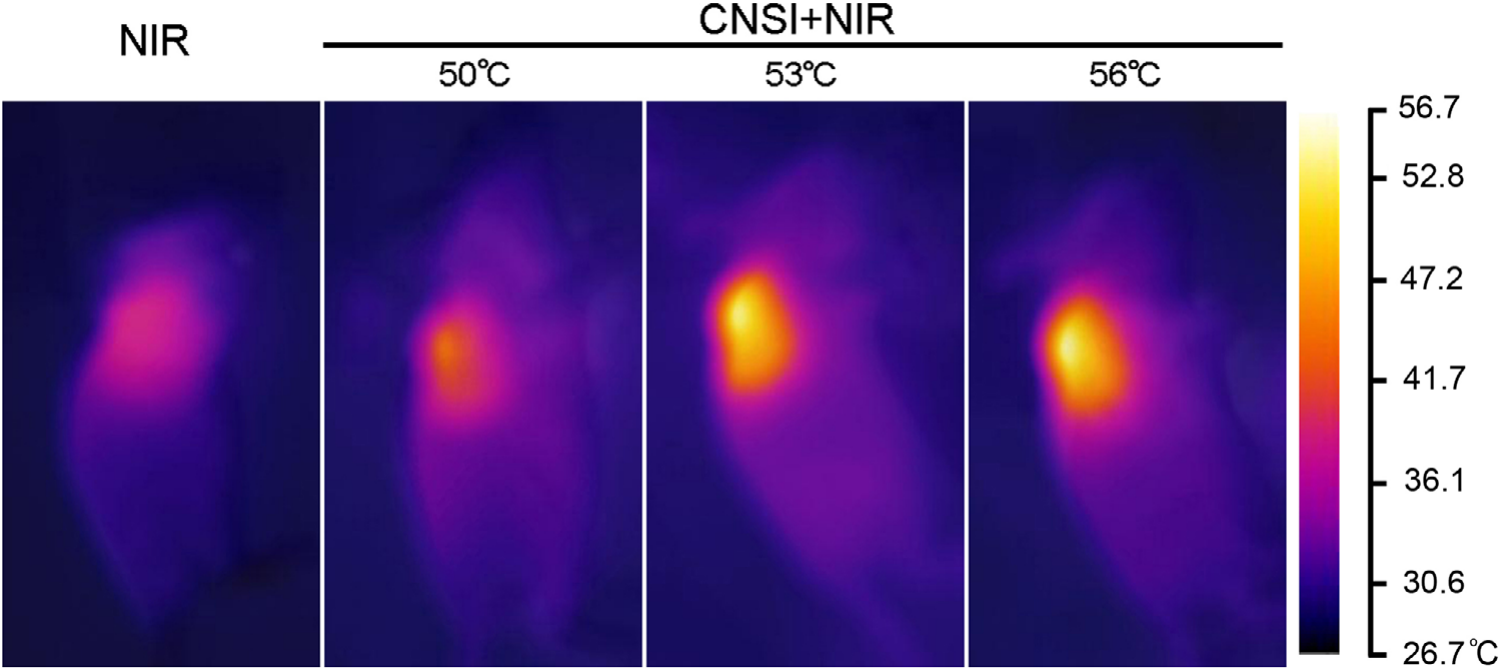
IR thermal images of tumor bearing mice under NIR irradiation with/without of CNSI injection (25 µL, 50 mg/mL)

The *in vivo* tumor ablation was achieved by intratumoral injection of CNSI followed by the NIR irradiation. Intratumoral injection rather than intravenous injection was adopted, because CNSI did not accumulate in tumor upon intravenous injection.^28^ The IR thermal imager recorded the temperature of the tumor sites (Figure 4). The NIR irradiation slightly increased the temperature of tumor site to 39.5^°^C. Using CNSI as a PTA, rapid temperature increases were achieved within the first 0.5 minute, and temperatures were maintained at optimal values for therapy. The temperatures of 50^°^C, 53^°^C, and 56^°^C were visualized by IR thermal imager, too.

**FIGURE 5:**
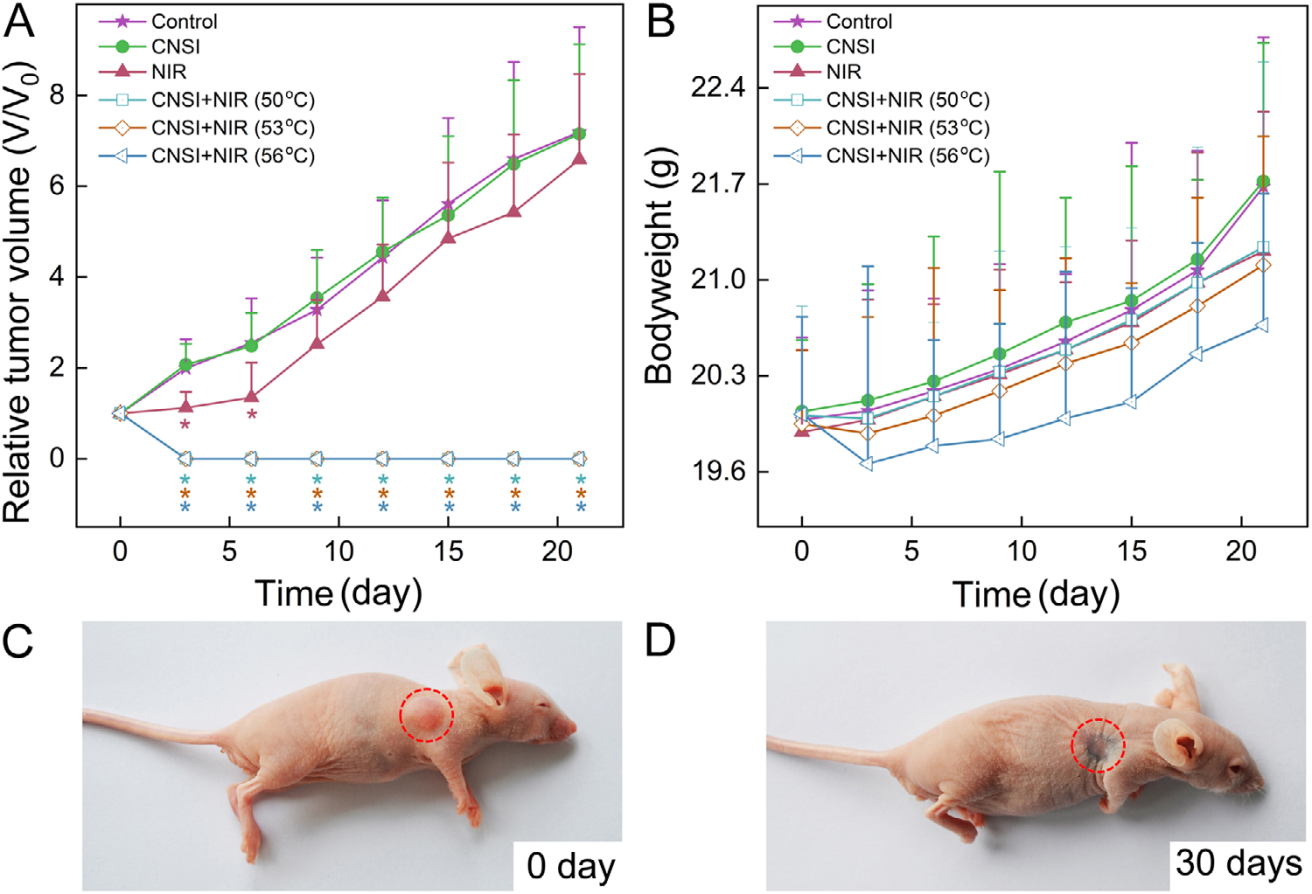
Tumor ablation by CNSI (25 µL, 50 mg/mL) under 808 nm laser irradiation: (A) tumor volume; (B) bodyweight; (C, D) photographs of CNSI+NIR (56^°^C) group before (C) and after (D) treatment. *P < .05 compared with control group (*n *= 8)

The efficient photothermal conversion capability of CNSI resulted in impressive PTT efficiencies in antitumor evaluations (Figure 5A). In the control group, the tumor volume steadily increased over time across the 21‐day observation period. The tumor growth pattern in the CNSI treated group without laser irradiation was nearly identical to the control group (Figure S6). The NIR irradiation without CNSI injection significantly slowed the tumor growth in the first 6 days, but the tumor growth displayed similar levels to control thereafter. In contrast, tumor ablation at 50^°^C to 56^°^C using CNSI as a PTA resulted in complete removal of the tumor. The tumor vanished and the volumes decreased to 0 mm^3^ (Figures 5C and D). This suggested that CNSI was a high‐performance PTA for tumor ablation. Beyond that, we continuously observed the mice up to 3 months. The tumors of CNSI+NIR (50^°^C) group recurred after 25 days. The tumors of CNSI+NIR (53^°^C) group and CNSI+NIR (56^°^C) group did not recur in 3 months. The recurrence results suggested that 53^°^C was required to avoid the tumor recurrence. The tumor cells were not completely killed at 50^°^C, which led to the recurrence. According to the bodyweight increases, there were slight decreases in the CNSI+NIR (56^°^C) group (Figure 5B). The rest groups showed similar bodyweight increases to that of the control group. Our results highlighted the importance of temperature in the PTT applications of carbon nanomaterials. In Frazier et al's study, they also observed the temperature‐dependent therapy of tumors using gold nanorod as the PTA.^29^ Similarly, Chen et al used conjugated polymers for photothermal tumor therapy, in which they observed higher tumor inhibition at irradiation power density of 1.0 W/cm^2^ (corresponding to a temperature increase of 16.5^°^C) than 0.5 W/cm^2^ (temperature increase of 5.2^°^C).^30^ Therefore, to achieve enhanced therapeutic efficiency, we concluded that future studies should precisely control the heating temperature during PTT.

**FIGURE 6:**
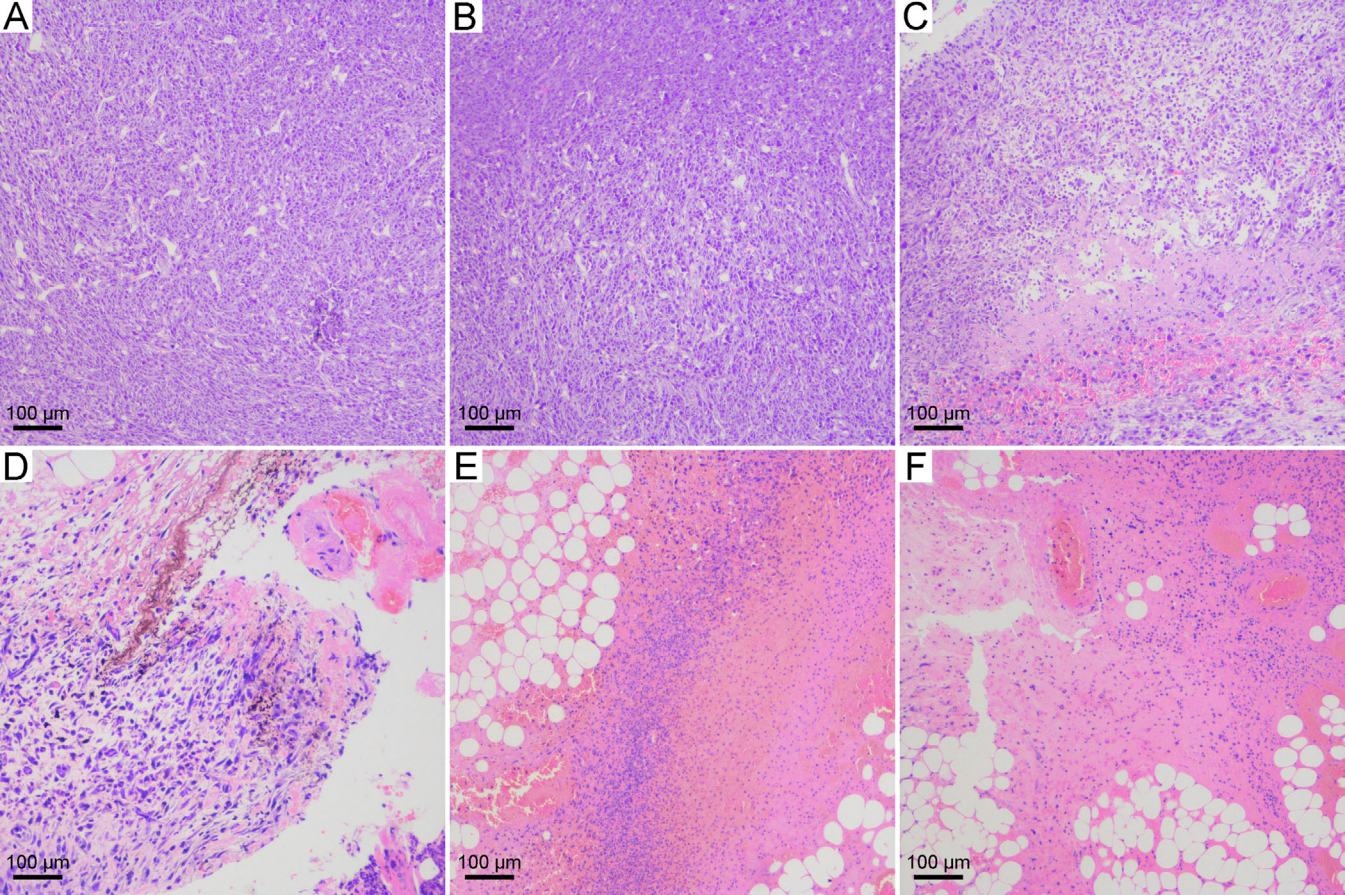
HE staining images of tumor tissues after the treatments: (A) control group; (B) CNSI group; (C) NIR group; (D) CNSI+NIR (50^°^C) group; (E) CNSI+NIR (53^°^C) group; and (F) CNSI+NIR (56^°^C) group

We checked the tumor tissues by HE staining at 2 days after the ablation (Figure 6). In the control group, the arrangement of tumor cells was loose and disordered. The karyotypes of tumor cells were polygonal. There was no obvious differentiation structure or nuclear division. The tumor cells in CNSI group without irradiation showed the same characteristics of the control. NIR irradiation alone induced slight necrosis of the tumor and some irregular cavities. The necrotic focus displayed the symptom of pyknosis, and was surrounded by coagulation‐like necrotic tissue residue and bleeding focus. The tumor ablation at 50^°^C with CNSI as the PTA resulted in long fusiform fibrous cells, focal necrosis, and local brownish‐yellow pigmentation. Meanwhile, following tumor ablation at 53^°^C, serious necrosis and bleeding were observed. A large number of necrotic cells and inflammatory cell infiltration were presented. In fact, it was hard to recognize the tumor cells due to the level of destruction. Very similar phenomena were found in the CNSI+NIR (56^°^C) group. Overall, the HE staining results suggested that PTT with CNSI efficiently destructed the tumor tissues.

Our results collectively indicated that CNSI had large absorption cross‐section, excellent photothermal conversion efficiency and low toxicity, making CNSI suitable for PTT of tumors. The PTT performance of CNSI was competitive to other carbon nanomaterials.^10^, ^11^, ^12^, ^26^, ^27^ Considering the two main issues hindering the clinical uses of carbon nanomaterials, CNSI had congenital advantages. First, CNSI is a commercially available medicine of large‐scale production, indicating the quality control of CNSI is reliable and the batch‐to‐batch variations are small. Second, the clinical applications on over 100 000 patients per year supported the good biosafety of CNSI. Beyond that, the intratumoral injection of CNSI is a well‐established technique for doctors. There is no need to functionalize CNSI by target moieties, which are expensive and complicated. Therefore, CNSI without any formulation change is hopeful to benefit the cancer patients through “off label” use in near future. In addition, CNSI is an excellent reagent to stain tumor drainage lymph nodes. Although we did not irradiate the lymph nodes in our study, in future applications, the laser irradiation could be applied to heat up the tumor drainage lymph nodes to inhibit the metastasis. Considering PPT is a general method for all tumors, CNSI could be applied in different cancer models.

For thyroid cancer in particular, the thyroid tumors (diameters of 1 cm or smaller) are generally confined in the capsule without draining lymph node metastasis or distant metastasis, making thyroid cancer an ideal condition for local treatment.^31^ Furthermore, thyroid cancer is a low‐malignant cancer that can lie dormant for a long time without further development.^32^ These characteristics make the clinicians look for a simpler and more effective technology as the alternative of surgery. Nowadays, the clinical practice of local radiofrequency hyperthermia for thyroid cancer of diameters within 1 cm is very common in China. However, the main disadvantage of this therapy is that the local hyperthermia is easy to damage the recurrent laryngeal nerve on the side of the thyroid, which leads to the hoarseness after treatment. In contrast, using CNSI as the PTA, the injury of recurrent laryngeal nerve would be avoided to a great extent, because CNSI does not stain the parathyroid gland.^16^, ^17^ Using this treatment, the thyroid tumor can be ablated by heat from the photothermal conversion of CNSI, while areas without CNSI accumulation do not experience these temperature increases. Thus, CNSI‐based PTT is extremely suitable for thyroid cancer therapy.

## 3 CONCLUSIONS

In summary, CNSI could efficiently convert NIR light into heat for PTT of tumors, where the tumor did not recur within the observation period. CNSI had strong absorbance in the NIR I window. Under NIR irradiation, CNSI could be heated up to kill cancer cells and ablate tumor tissues. Taking the good biocompatibility, low toxicity, and great success in clinical applications of CNSI in consideration, clinical applications of CNSI in PTT through “off label” use should be urgently evaluated to benefit the cancer patients. We hope that our results would stimulate more research interests and clinical applications of CNSI in theranostics.

## 4 EXPERIMENTAL SECTION

### 4.1 Materials

The starting material CH40 was bought from Mitsubish Chemical Co., Tokyo, Japan, and characterized by SEM (JSM‐7500, JEOL, JEOL, Japan), XPS (Axis Ultra, Kratos, Manchester, UK), and Raman spectroscopy (inVia, Renishaw, New Mills, UK). CNSI was provided by Chongqing Lummy Pharmaceutical Co., Ltd., Chongqing, China. CNSI was characterized UV‐vis‐NIR spectrometer (U‐4100, Hitachi, Tokyo, Japan), DLS (Zerasizer Nano ZS90, Malvern, Malvern, UK), XRD (XD‐6, Purkinje General Instrument Co., Beijing, China), IR (Magna‐IR 750, Nicolet, Madison, USA), and TEM (H‐600IV, Hitachi, Tokyo, Japan) by before use. The 0.9% sodium chloride injection was bought from Sichuan Kelun Pharmaceutical Co., Ltd., Chengdu, China. RPMI‐1640 medium, cell digestive trypsin, penicillin streptomycin mixture, and phosphate buffer (PBS) were obtained from Hyclone Co., Logan, USA. Fetal bovine serum was bought from Gibco Co., New York, USA. TCP‐1 thyroid cancer cells were kindly provided by the Department of Thyroid Surgery, West China Hospital of Sichuan University. BALB/c‐nu nude mice were bought from Chengdu Dashuo Biotechnology Co., Ltd., Chengdu, China.

### 4.2 Heating of CNSI *in vitro*

CNSI of different concentrations (5 to 500 µg/mL) was placed in glass dishes (1.0 mL/dish). The dishes were irradiated under NIR laser (808 nm, CW laser) at 0.5, 1.0, and 2.0 W/cm^2^ for 5 minutes. The initial temperature and the temperature changes upon irradiation were recorded on a thermometer (opSens, Tempsens Instrument Pvt. Ltd., Québec, Canada). The blank solution without CNSI was treated the same way. The DLS spectra of CNSI after different irradiation times were recorded to monitor the particle size changes.

### 4.3 Hyperthermia of thyroid cancer cells by CNSI *in vitro*

Thyroid cancer TPC‐1 cells were cultured following the literature protocol. To a glass dish, 1.0 mL cell suspension (3 × 10^4^ cells/mL) was added. The dishes were incubated at 37°C under 5% CO_2_ flow for 24 hours. Then, the medium was discharged and replaced by new culture medium supplemented with 50 µg/mL of CNSI. After another 24‐hour incubation, the dishes were irradiated by 808 nm laser at 0.5 W/cm^2^ for 3 minutes. During the irradiation, the distance between laser and the dish was adjusted to maintain the temperature at 48°C to 56°C guided by the IR thermal imager (Ti400, Fluke, Everett, USA). After irradiation, the culture medium was renewed. The irradiation treatment was repeated two more times at an interval of 48 hours. After the third irradiation, the cells were digested with trypsin for counting. For the control group, no CNSI was supplemented to the medium and no irradiation was applied. For CNSI group, no irradiation was applied. For NIR group, no CNSI was supplemented to the medium, but the irradiation was applied and the distance between laser and the dish was set at 15 cm. In another set of experiments, the cell viabilities were measured with the supplement of CNSI (5 to 100 µg/mL) in the presence/absence of NIR irradiation.

### 4.4 Thermal ablation with CNSI *in vivo*

The animal experiments were approved by the Institutional Animal Care and Use Committee of Southwest Minzu University and performed in compliance with the Animal Care and Use Program Guidelines of the Sichuan Province, China. Animals were housed in plastic cages (5 mice/cage) and kept on a 12‐hour light/dark cycle. Food and water were provided *ad libitum*. Following acclimation for 7 days, the mice were inoculated by injection of 0.1 mL TPC‐1 cell suspension (3 × 10^6^ cells) on the right limb. The thermal ablation experiments were performed when the tumor sizes reached 100 to 150 mm^3^. The mice were randomly divided into six groups, namely, control group, CNSI group, NIR group, and three CNSI+NIR groups differing in their treatment temperature (50°C, 53°C, and 56°C separately for each group). The mice were intratumorally injected with 25 µL of CNSI. After 5 minutes, the tumor was irradiated by 808 nm laser at 0.5 W/cm^2^ for 3 minutes. During the irradiation, the distance between laser and the dish was adjusted to maintain the temperature at 50°C, 53°C, and 56°C guided by the IR thermal imager. The irradiation was repeated two additional times at an interval of 24 hours. For the control group, no CNSI was supplemented to the medium and no irradiation was applied. For CNSI group, no irradiation was applied. For NIR group, no CNSI was injected, but the irradiation was applied and the distance between laser and the dish was set as 15 cm. The tumor size and bodyweight were recorded with intervals of 2 days. Two of each group were sacrificed for HE staining. The tumor tissues were dissected, fixed with 10% formaldehyde, and sampled following the standard protocol of HE staining (Eclipse ci, Nikon, Tokyo, Japan). The rest eight of each group were observed for 3 months.

### 4.5 Statistical analysis

The data of cell and animal experiments were analyzed as the mean with the standard deviation (mean ± SD). The significance was calculated by Student's t‐test. Differences were considered significant at *P* < 0.05.

## CONFLICT OF INTEREST STATEMENT

YH, GZ, QX, JY, CZ, and XT are the employees of Sichuan Enray Pharmaceutical Sciences Company.

## AUTHOR CONTRIBUTION STATEMENT

STY and XT conceived and designed the research. STY and XT cowrote the paper. YH and GZ performed the animal evaluations. QX and CZ prepared the CNSI samples and characterized them. JY performed the cell experiments. KT is a research participant from Chengdu No. 7 High School, who was involved in Raman analysis. All authors discussed the results and commented on the manuscript.

## Supporting information


**Supporting Information**: Additional supporting information may be found online in the Supporting Information section at the end of the article.
