# Supplementary material for: Carbon nanoparticles suspension injection for photothermal therapy of xenografted human thyroid carcinoma in vivo
Source: MedComm (2020). 2020 Sep 10;1(2):202–10. doi: 10.1002/mco2.28 (PMC8491229; doi:10.1002/mco2.28)
Supplement: Supplementary file 1 — Supporting Information: Additional supporting information may be found online in the Supporting Information section at the end of the article. [file MCO2-1-202-s001.docx]

**Carbon nanoparticles suspension injection for photothermal therapy of xenografted human thyroid carcinoma** ***in vivo***

Yuanfang Huang,^1^ Guangfu Zeng,^1^ Qian Xin,^1^ Jinmei Yang,^1^ Cheng Zeng,^1^ Kexin Tang,^2^ Sheng-Tao Yang,^2,*^ & Xiaohai Tang^1,*^

^1^ Sichuan Enray Pharmaceutical Sciences Company, Chengdu 610041, P. R. China.

^2^ College of Chemistry and Environment Protection Engineering, Southwest Minzu University, Chengdu 610041, P. R. China.

* To whom correspondence should be addressed: Prof. Sheng-Tao Yang (email address: [yangst@pku.edu.cn](mailto:yangst@pku.edu.cn)) and Prof. Xiaohai Tang (email address: [pharmmateceo@enraypharm.com](mailto:pharmmateceo@enraypharm.com)).

Number of pages: 5

Number of figures: 6

**Supporting Information**

**Supplementary figures**


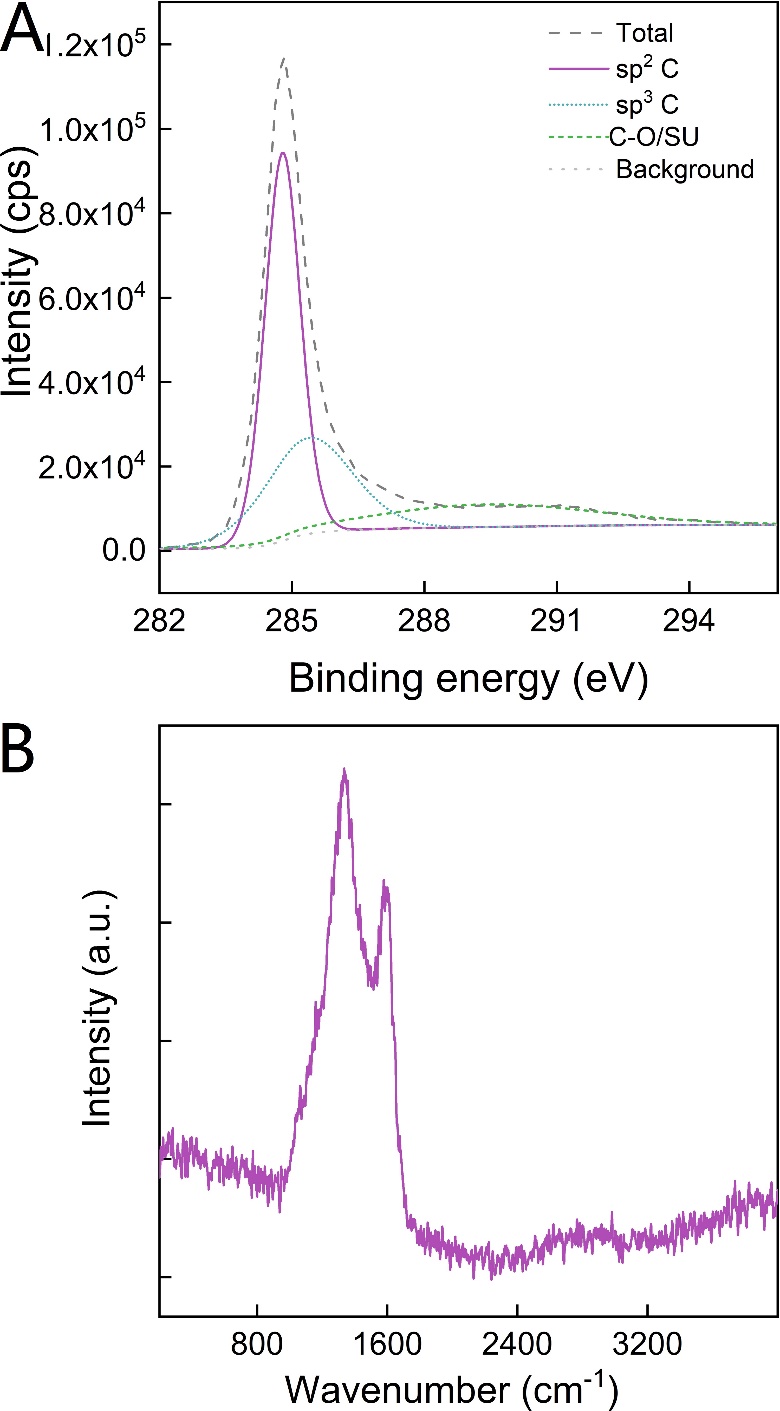


**Figure S1.** Characterization of CH40 ash. (A) C1s XPS spectrum; (B) Raman spectrum.


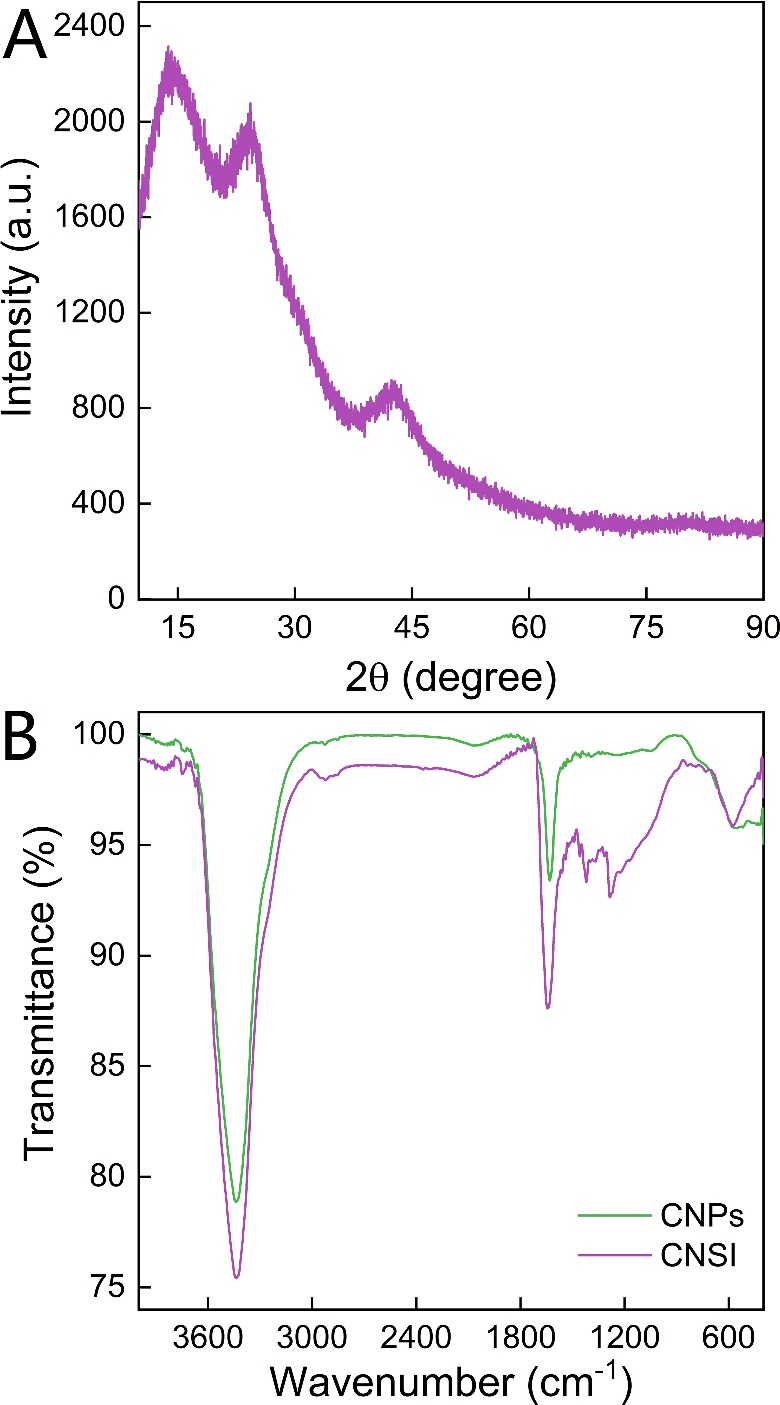


**Figure S2.** Characterization of CNSI. (A) XRD spectrum of CH40 ash; (B) IR spectra of CH40 ash (CNPs) and CNSI.


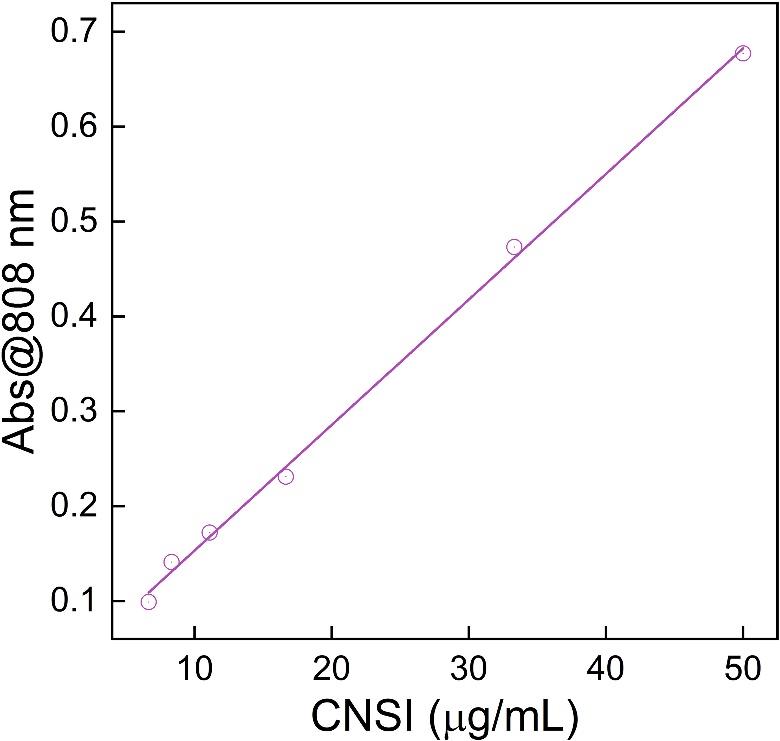


**Figure S3.** Standard calibration line of the absorbance@808 nm versus the concentration of CNSI.


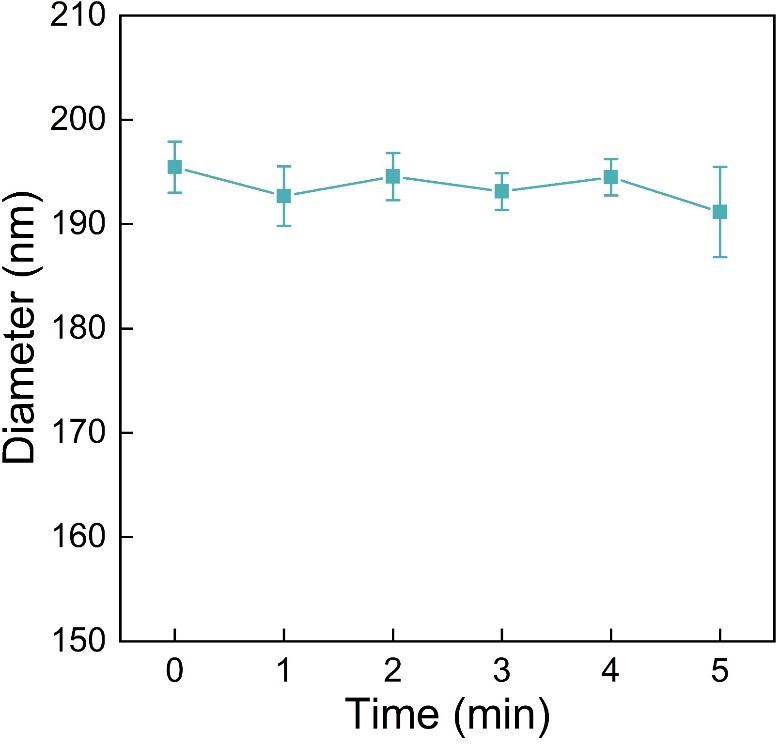


**Figure S4.** Particle size changes of CNSI upon laser irradiation.


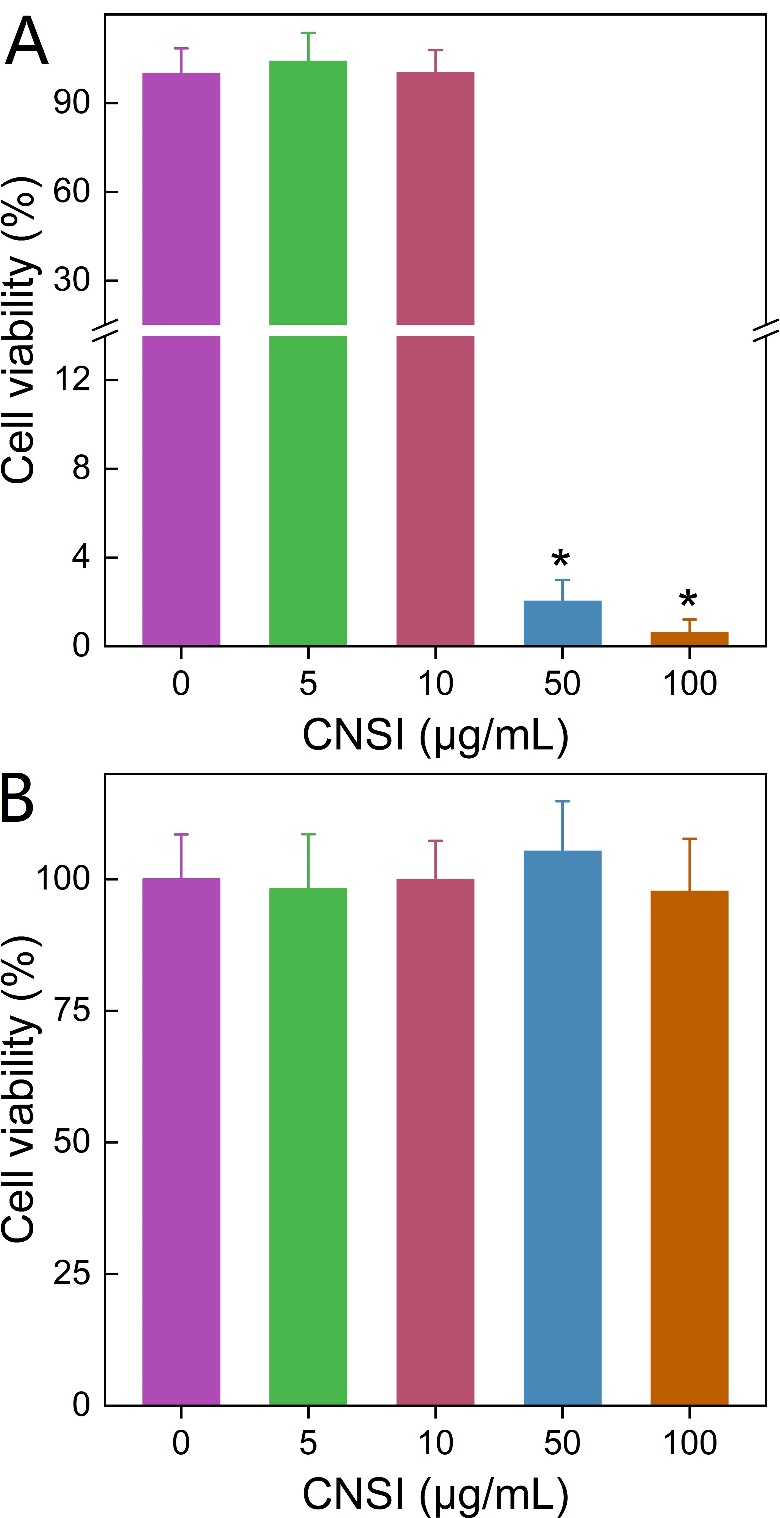


**Figure S5.** Cell viability of TPC-1 cells after CNSI+NIR treatment (A) and CNSI alone exposure (B) at different concentrations. * *p* < 0.05 compared with control group (*n*=3).


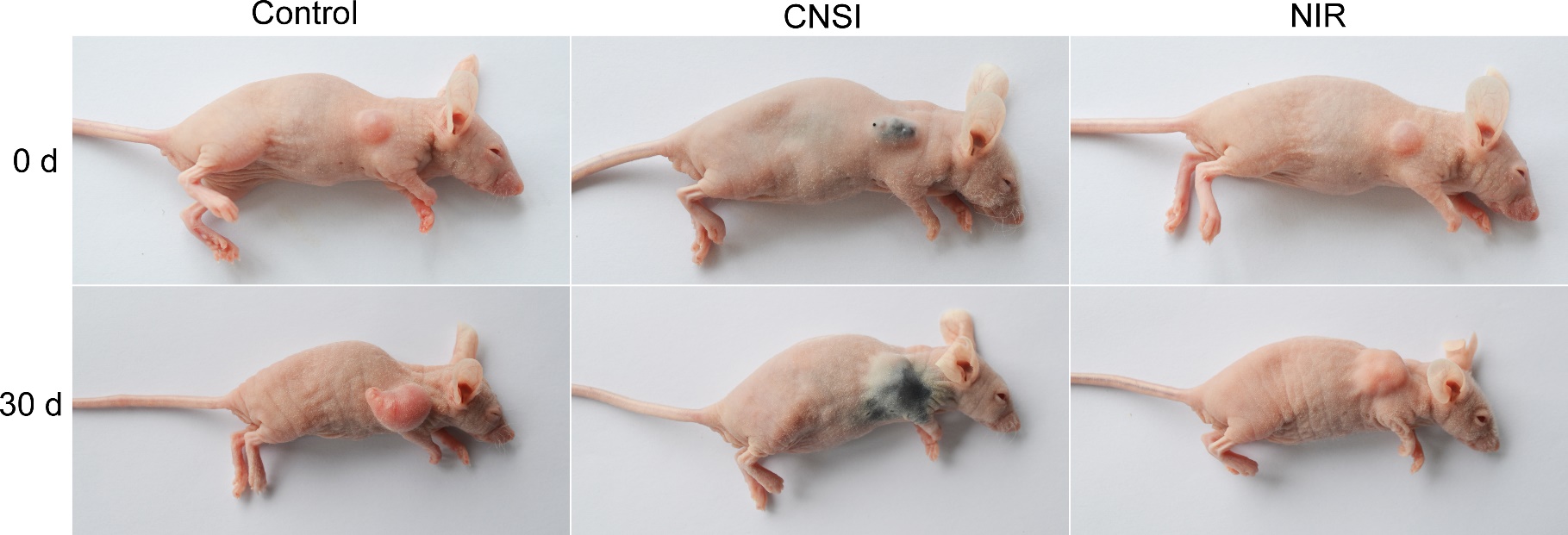


**Figure S6.** Photographs of mice before and after the treatment.
